# Supernumerary ectopic tooth on the maxillary sinus

**DOI:** 10.11604/pamj.2014.18.353.4912

**Published:** 2014-08-31

**Authors:** Nabil Touiheme, Abdelhamid Messary

**Affiliations:** 1 Department of ENT. Military Hospital Moulay Ismail, Morocco

**Keywords:** Ectopic tooth, maxillary sinus, recurrent sinusitis

## Image in medicine

A 23 year-old-woman was referred to point out for pain on the left side of her face and mucopurulent rhinorrhoea lasting for 07 months with long history of recurrent sinusitis. Antibiotics and pain-killers were prescribed to him in the first time. On examination of the oral cavity, all the permanent teeth were present. At nasal endoscopic examination we found a mucopus trickling from the left middle meatus.clinical diagnosis is for sinusitis of dental origin, fungal sinusitis or chronic rhinosinusitis Coronal computed tomography (CT) of the paranasal sinuses revealed the presence of a supernumerary molar tooth (Arrow) within left maxillary sinus floor (A). The tooth was extracted by traditional approach (Caldwell-Luc procedure) from the maxillary sinus under general anesthesia (B, C, D), and the patient has been asymptomatic for more than 02 years. The presence of ectopic or supernumerary tooth in the maxillary sinus is most often asymptomatic but on rare occasions can be a cause of recurrent sinusitis. The supernumerary ectopic tooth is a very rare condition, usually it's localized on the maxillary sinus, and other sites were described like nasal cavity or chin. Coronal computed tomography is sufficient to determine extract localization. The extraction can be made by conventional approach or by transnasal endoscopic approach with less morbidity.

**Figure 1:**
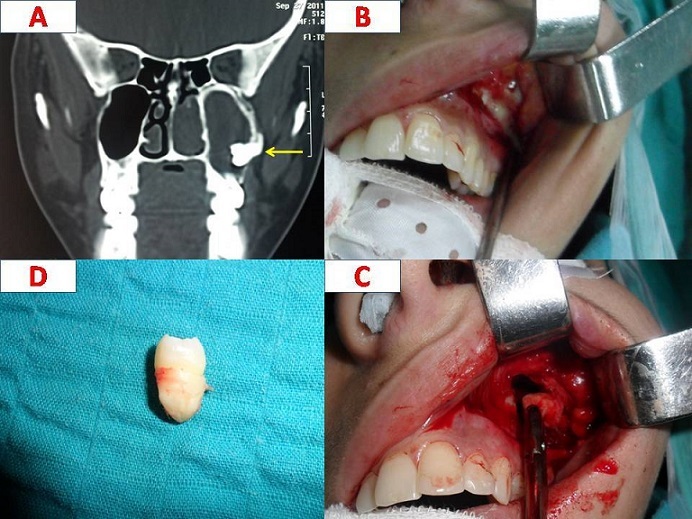
A): A coronal computed tomography (CT) of the paranasal sinuses in bone window revealing a filling of the left maxillary sinus byanhyperdense process attached to the outer wall: ectopic tooth; B): An operative view demonstrated the presence of an ectopic and supernumerary tooth in the maxillary sinus after incision according to Caldwell-Luc procedure; C): An operative view showing the extraction of the toothon the maxillary sinus; D): The extracted supernumeraryand ectopic tooth

